# COVID-19 Compared to Other Pandemic Diseases

**DOI:** 10.5041/RMMJ.10418

**Published:** 2020-07-31

**Authors:** Silvio Daniel Pitlik

**Affiliations:** Infectious Diseases, Mayanei Hayeshua Medical Center, Bnei Brak, Israel

**Keywords:** COVID-19, comparison, history, pandemics, SARS-CoV-2

## Abstract

In December 2019, the first cases of a new contagious disease were diagnosed in the city of Wuhan, the capital of Hubei province in China. Within a short period of time the outbreak developed exponentially into a pandemic that infected millions of people, with a global death toll of more than 500,000 during its first 6 months. Eventually, the novel disease was named coronavirus disease 2019 (COVID-19), and the new virus was identified as severe acute respiratory syndrome coronavirus 2 (SARS-CoV-2). Similar to all known pandemics throughout history, COVID-19 has been accompanied by a large degree of fear, anxiety, uncertainty, and economic disaster worldwide. Despite multiple publications and increasing knowledge regarding the biological secrets of SARS-CoV-2, as of the writing of this paper, there is neither an approved vaccine nor medication to prevent infection or cure for this highly infectious disease. Past pandemics were caused by a wide range of microbes, primarily viruses, but also bacteria. Characteristically, a significant proportion of them originated in different animal species (zoonoses). Since an understanding of the microbial cause of these diseases was unveiled relatively late in human history, past pandemics were often attributed to strange causes including punishment from God, demonic activity, or volatile unspecified substances. Although a high case fatality ratio was common to all pandemic diseases, some striking clinical characteristics of each disease allowed contemporaneous people to clinically diagnose the infection despite null microbiological information. In comparison to past pandemics, SARS-CoV-2 has tricky and complex mechanisms that have facilitated its rapid and catastrophic spread worldwide.

## INTRODUCTON

On January 10, 2020, the genome of a new coronavirus, now known as severe acute respiratory syndrome coronavirus 2 (SARS-CoV-2), was posted on the internet.^1^ It had been isolated days before from patients developing varying degrees of pneumonia in Wuhan, the capital of Hubei province in China.^2^ Immediately thereafter, a growing number of scientists worldwide became deeply involved in analyzing its molecular details.^3^ One of the major tasks that they focused on was synthetizing the proteins encoded by the viral RNA and deciphering their structure and function. Acutely aware of the pandemic potential of SARS-CoV-2, some of these scientists immediately alerted selected vaccine producers, with the hope of triggering a swift process for vaccine design and development.^4^,^5^ Once the protein amino acid composition and the post folding structure of close to 30 proteins in SARS-CoV-2 were defined, multiple computational searches were launched by a number of institutions, looking to repurpose extant drugs aimed against the newly discovered molecular targets.^6^ In parallel to this accelerated research, there was a marked and frightening spread of this new coronavirus throughout Wuhan to a widening area in China, and it was subsequently exported to a rapidly growing list of countries worldwide.^7^,^8^

This paper reviews the microbiological, clinical, and epidemiological characteristics of the coronavirus disease 2019 (COVID-19) pandemic, as well as its socio-economic impact. In addition, COVID-19 is compared to previous pandemics in human history.

## UNDERSTANDING SARS-COV-2

In the early days of the pandemic great effort was invested into understanding the life cycle of SARS-CoV-2,^9^ so as to provide a basis for discovery of an effective vaccine to prevent COVID-19 and/or a safe and efficacious drug to cure it, or at the least, to ameliorate its symptoms, shorten its duration, and/or block its mechanism of transmission. Being a virus, SARS-CoV-2 must invade host cells, hijack the cell’s biologic machinery for reproduction, and, finally, release multiple daughter virions. Research uncovered six steps in the life cycle of SARS-CoV-2: (1) attachment and entry; (2) uncoating; (3) guide ribonucleic acid (gRNA) replication; (4) translation in the endoplasmic reticulum and Golgi apparatus; (5) assembly; and (6) virion release.^10^,^11^

The external surface of SARS-CoV-2 has multiple protruding elements called spike proteins which, after manipulation by host cell enzymes (furin and TMPRSS2), function as anchors for attachment to the host cells.^12^,^13^ The cell surfaces of the upper and lower respiratory tract are covered with angiotensin-converting enzyme-2 (ACE2) receptors, which are physiologically involved in blood pressure regulation.^13^ However, these receptors are also present in many other organs and tissues, helping to explain some of the extra-respiratory manifestations of COVID-19.^14^ Once attached to the external membrane, SARS-CoV-2 covers itself with a portion of the host cell membrane and becomes an intracellular endosome. This structure undergoes partial uncoating allowing the release of gRNA into the cytoplasm of the host cell. The released strands of gRNA attach to host ribosomes, RNA-dependent RNA polymerase (RdRp), and together activate the gRNA replication mechanism. Other released strands of gRNA undergo translation into structural, non-structural, and coat proteins.^9^ The different basic blocks of the reproduced virus are finally assembled into multiple virions that are expelled to the extracellular space of the host.^9^ These released daughter virions are now ready to infect other cells or, even worse, other hosts.

Once these mechanisms had been clarified, multiple hypotheses relating to specific actions of different drugs were proposed. For example, chloroquine, hydroxychloroquine, and azithromycin all inhibit the uncoating of the invading endosomes. On the other hand, the antivirals remdesivir and favipiravir inhibit gRNA replication by RdRp. Additional drugs, not only antivirals, have also been identified to target the complex mechanisms of the intracellular viral cycle.^15^

## ANIMAL AND HUMAN CORONAVIRUS

The first human coronavirus was described by June Dalziel Almeida in 1966. She had observed a viral structure seen under electron microscopy while being involved in a study investigating causes of the common cold. A paper submitted by Almeida and her team described a crown-shaped structure supposed to be a new type of virus causing common colds. This paper was rejected as the editors claimed that “these microscopic observations resulted from distorted influenza viruses.”^16^,^17^ Since this pioneering observation, around 100 species of viruses in the subfamily Coronaviridae have been described, the majority of which were found in animals, primarily though not only in bats.^18^ Only some of the animal coronaviruses had been associated with a specific animal disease such as a severe type of bronchitis in poultry.^17^ On the other hand, many of these coronaviruses were isolated from healthy animals, again predominantly bats.

**Figure 1:**
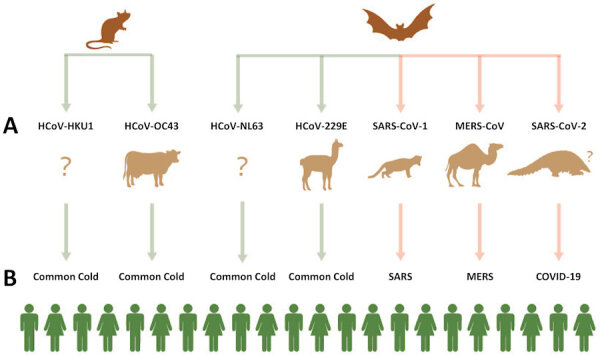
Zoonotic Origin of Human Coronaviruses (A) and the Resulting Diseases (B) Upper line, reservoir hosts; middle line, intermediate hosts; bottom line, infected human hosts. COVID-19, coronavirus disease 2019; HCoV-229E, human coronavirus 229E; HCoV-NL63, human coronavirus Netherlands 63; HCoV-OC43, human coronavirus OC43; HCoV-HKU1, human coronavirus Hong Kong U1; MERS, Middle East respiratory syndrome; MERS-CoV, Middle East respiratory syndrome coronavirus; SARS, severe acute respiratory syndrome; SARS-CoV-1, severe acute respiratory syndrome coronavirus-1; SARS-CoV-2, severe acute respiratory syndrome coronavirus-2.

Based on current knowledge, there are seven species of human coronaviruses.^19^ Four of them (Figure 1, Table 1) cause a mild upper respiratory tract infection manifested as a runny nose. Occasionally, they involve the lower respiratory tract. The remaining three human coronaviruses are associated with a wider spectrum of disease severity. Relatively frequently they cause severe pneumonia and other serious complications (Figure 1, Table 1). As indicated in Figure 1, five of the seven human coronaviruses have well established intermediate hosts, based on both epidemiologic and genomic data. In the case of COVID-19, preliminary data suggest that several species of pangolins are the suspected intermediate host. Pangolins are mammals covered by keratin scales. In China the pangolin is seen as an edible animal mainly, but traditional medicine in this country also attributes multiple curative properties to a powder obtained by mashing their scales.^20^,^21^

**Table 1:** Known Human Diseases Caused by a Coronavirus (see also Figure 1).

Disease	Virus	Main Manifestation(s)	Hospitalization	Antiviral Therapy	Vaccine	Case Fatality (%)
Common cold	HCoV-HKU1	Runny nose*	Very rare	None	None	0
Common cold	HCoV-OC43	Runny nose*	Very rare	None	None	0
Common cold	HCoV-NL63	Runny nose*	Very rare	None	None	0
Common cold	HCoV-229E	Runny nose*	Very rare	None	None	0
SARS	SARS-CoV-1	Pneumonia	Very frequent	None	None	9.5
MERS	MERS-CoV	Pneumonia	Very frequent	None	None	34.4
COVID-19	SARS-CoV-2	Respiratory tract infection†	Frequent	Remdesivir	None‡	>1.6§

* Rare: lower respiratory tract infection (bronchitis or pneumonia).

† Upper respiratory tract infection frequent, pneumonia in a minority of patients.

‡ 125 different vaccines in various phases of development, none yet approved.

§ Case fatality value varies with population characteristics, age distribution, quality of healthcare services, medical equipment, etc.

COVID-19, coronavirus disease 2019; HCoV-229E, human coronavirus 229E; HCoV-HKU1, human coronavirus Hong Kong U1; HCoV-NL63, human coronavirus Netherlands 63; HCoV-OC43, human coronavirus OC43; MERS-CoV, Middle East respiratory syndrome coronavirus; SARS-CoV-1, severe acute respiratory syndrome-coronavirus-1; SARS-CoV-2, severe acute respiratory syndrome coronavirus-2.

Although the transmissibility of both severe acute respiratory syndrome (SARS) and Middle East respiratory syndrome (MERS) is lower than that of COVID-19, the case fatality ratio is many times higher for MERS and SARS than for COVID-19 (Table 1).^22^ However, the number of COVID-19 cases have markedly outnumbered the number of cases in both SARS and MERS. While clinical manifestations for the two last-mentioned infections were generally limited to the respiratory tract, although with higher degrees of disease severity, it is remarkable that there has been a wide spectrum of heterogeneous clinical manifestations in COVID-19 cases. From a pathophysiological perspective, this phenomenon is explained by the ubiquitous presence of ACE2 receptors throughout multiple organs and blood vessels. At a clinical level, some typical COVID-19 manifestations described during the pandemic include “silent anoxia,”^23^ a discrepancy between an extremely low oxygen level as measured by pulse oximeter and the simultaneous lack of dyspnea; signs of cardiac involvement including myocarditis, myocardial ischemia, and myocardial infarction^24^; hepatitis^25^; reddish discoloration of the toes mimicking frostbite or chilblains^26^; intravascular coagulation including pulmonary embolism^27^; encephalitis^28^; and acute renal failure (see also Table 2).^29^ Recently a condition similar to Kawasaki syndrome was described in a growing number of teenagers (as opposed to toddlers with classical Kawasaki syndrome); it has been proposed to name the syndrome “pediatric multisystem inflammatory syndrome.”^30^ The mechanism of this complication is an overreaction of the immune system. A comparison of the leading clinical identifiers of recognized pandemic diseases is provided in Table 2.

**Table 2:** Leading Clinical Identifiers of Recognized Pandemic Diseases.

Disease	Salient Clinical Features
Smallpox	Typical widespread vesiculo-pustular rash, occasionally corneal opacification
Measles	Morbilliform rash, Koplik’s spots, conjunctivitis, rhinorrhea
Plague	Buboes (huge lymphadenopathy), pneumonia
Cholera	Sudden-onset profuse watery diarrhea, early hypovolemic shock
Yellow fever	Jaundice
Influenza H1N1	Flu-like illness*, severe disease and death in young adults
Influenza H3N2	Flu-like illness*
Influenza H2N2	Flu-like illness*
AIDS	Opportunistic infections, Kaposi’s sarcoma of skin and viscera, profound emaciation
SARS	Severe pneumonia
Ebola	Bleeding from multiple sites
MERS	Severe pneumonia
COVID-19	Severe pneumonia, silent anoxia, anosmia, ageusia, toe lesions mimicking chilblains, pediatric multisystem inflammatory syndrome

* Fever, myalgia, respiratory symptoms, extreme weakness.

AIDS, acquired immune deficiency syndrome; COVID-19, coronavirus disease 2019; Flu, influenza; H1N1, hemagglutinin-1 neuraminidase-1; H2N2, hemagglutinin-2 neuraminidase-2; H3N2, hemagglutinin-3 neuraminidase-2; MERS, Middle East respiratory syndrome; SARS, severe acute respiratory syndrome.

## DEFINITION OF PANDEMICS

The simplest definition of a pandemic is a contagious infectious disease that has spread to multiple geographic areas or continents. The term “contagious” implies that the infection can be transmitted person-to-person, either directly or indirectly. Various degrees of controversy emerge between members of the medical and scientific communities when defining a new disease as pandemic. According to the World Health Organization (WHO), “a pandemic is the worldwide spread of a new disease.”^31^ However, even this condensed and crisp definition, not infrequently, leaves room for discussion. A 2009 convention, held at the beginning of the H1N1 pandemic under the umbrella of the National Institutes of Health, was asked to develop a more detailed definition of pandemics. This gathering of infectious diseases specialists and epidemiologists proposed eight characteristics of pandemics (Table 3).^32^ Some of these characteristics relate to the microbe itself, while others are dependent on interaction between the microbe and the involved human population. A large number of books dealing with pandemics have been published, mainly during this last decade.^32^–^38^ Many web sites^39^–^44^ and review articles have also reviewed the history of pandemics. Interestingly, there is a significant discordance in the inclusion or the exclusion of specific infectious diseases causing pandemics. Table 4 provides a chronological list of the major known pandemics reviewed in this article.^32^–^44^

**Table 3:** Eight Characteristics of Pandemics. The eight characteristics are related to the microbes themselves, or to the human–microbe interaction.*

Feature	Comments
Novelty	Mostly unknown to the medical profession
Minimal population immunity	Frequent absence of specific IgG antibodies
Explosiveness	Determined mainly by size or density of population and factors related to type of transmission, for example vector population
Fast disease movement	Type and speed of human transmission
Wide geographic extension	Social interaction of populations, widespread common source
Infectiousness	Ability of microbes to produce disease (minimal infective dose)
Contagiousness	Proportion of completely asymptomatic cases, super-spreaders, and evident and pathognomonic disease markers
Severity	Need for hospitalization, artificial ventilation, or intensive rehydration; chronicity or death

* Examples of interacting human factors include background immunity, means of transmission, and healthcare system quality.

**Table 4:** Chronology of Known Pandemics.*

Time	Name	Microbe	Death Toll
430 BC	The plague of Athens	*Rickettsia* spp? *Salmonella enterica* spp?	25% of population
165–180	Antonine plague	Smallpox? measles?	5M
541–542	Plague of Justinian	*Yersinia pestis* (Gram-negative bacteria)	30–50M
735–737	Japanese smallpox epidemic	Smallpox (DNA virus)	1M
1347–1351	Black death	*Yersinia pestis*	200M
1520-onward	New world smallpox	Variola (smallpox)	56M
1629–1631	Italian plague	*Yersinia pestis*	1M
1665–1666	Great plague of London	*Yersinia pestis*	100K
1800s†	Yellow fever	Yellow fever (RNA virus)	100–150K
1817–1923	Cholera pandemics	*Vibrio cholera* (Gram-negative bacteria)	>1M
1885	Third plague	*Yersinia pestis*	12M
1889–1890	Russian flu	Influenza H2N2? (RNA virus)	1M
1918–1919	Spanish flu	Influenza H1N1	40–50M
1957–1958	Asian flu	Influenza H2N2	1.1M
1968–1970	Hong Kong flu	Influenza H3N2	1M
1981–present	AIDS	HIV (RNA virus)	25–35M
2002–2003	SARS	SARS-CoV-1 (RNA virus)	0.8K
2009–2010	Swine flu	Influenza H1N1	200K
2014–2016	Ebola	Ebola virus (RNA virus)	11K
2015–present	MERS	MERS-CoV (RNA virus)	0.8K
2019–present	COVID-19	SARS-CoV-2 (RNA virus)	>0.5M

* Due to the lack of clear-cut definition of a pandemic, this table was compiled based on commonalities in multiple references.^32^–^44^

† There were more than 20 waves of Yellow fever in the 1800s; hence, a precise end-date cannot be given.

AIDS, acquired immune deficiency syndrome; BC, before Christ; COVID-19, coronavirus disease 2019; DNA, deoxyribonucleic acid; flu, influenza; H1N1, hemagglutinin-1 neuraminidase-1; H2N2, hemagglutinin-2 neuraminidase-2; H3N2, hemagglutinin-3 neuraminidase-2; HIV, human immune deficiency virus; K, thousands; M, millions; MERS, Middle East respiratory syndrome; MERS-CoV, Middle East respiratory syndrome coronavirus; RNA, ribonucleic acid; SARS, severe acute respiratory syndrome; SARS CoV-1, severe acute respiratory syndrome coronavirus-1; SARS CoV-2, severe acute respiratory syndrome coronavirus-2.

## HISTORY OF PANDEMICS

Obviously, written testimony on possible pandemics is lacking with regard to prehistoric times. However, it is important to recall that during the pre-agricultural era, the nomadic population of *Homo sapiens* on Earth was relatively small. Consequently, we can assume that pandemics were relatively rare at that time. The known history of pandemics is based on the discoveries of documentation by ancient historians and other sources. Table 4 provides an overview of the known pandemics throughout history.^32^–^38^ Due to the lack of consensus on the definition of a pandemic, there are some cases in which there is a discrepancy regarding the categorization of an epidemic as a pandemic. For example, the SARS epidemic in 2002–2003 had all the characteristics of a potential dire pandemic, but fortunately its spread was interrupted by yet unexplained mechanisms. Some pandemics such as AIDS have been widespread both in time and in vast geographic distribution.^45^ Current knowledge marks the 1930s as the beginning of the human immune deficiency virus (HIV) epidemic, when zoonoses from *Pan troglodytes troglodytes* to humans occurred in Africa.^46^,^47^ Similarly, the cholera and plague pandemics had a zoonotic origin. 48,49

**Figure 2:**
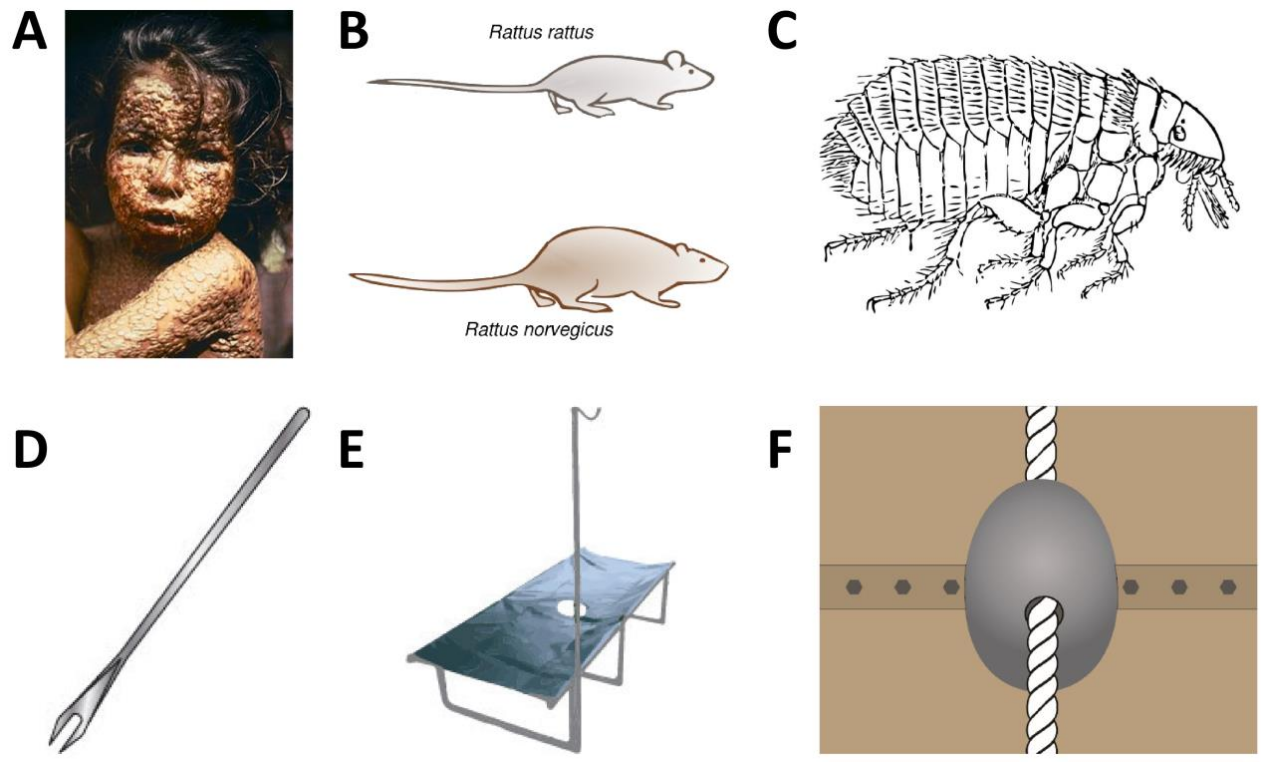
Illustrative Examples of Factors Affecting Spread (Top Row) or Control (Bottom Row) of Pandemics **A:** Typical smallpox rash with extremely contagious pustular lesions. **B:** The two most common species of rats (reservoirs of *Yersinia pestis*). **C:** *Xenopsylla cheopis* (the rat flea), one of the vectors of *Yersinia pestis*, which transmits the infecting bacteria from rats to human beings. **D:** Bifurcated needle used for smallpox vaccination during the last decades of smallpox eradication. **E:** Cholera bed used to rehydrate patients with severe diarrhea; the drain in the middle is used to facilitate drainage of the copious diarrheal fluid. **F:** Cone barrier used to prevent rats from invading or leaving ships through the mooring ropes of the ship. Panel A from Wikipedia.com, Public Domain by CDC/James Hicks; Panel B modified from Wikipedia.com (CC BY-SA 3.0); Panel C from Pixabay.com.

Figure 2 provides some illustrative examples of the human (panel A) and zoonotic (panels B and C) factors contributing to the spread of pandemics, and devices developed to help avoid their spread (panels D, E, and F).

Intriguingly, and according to databases managed by both Johns Hopkins University^7^ and the European Centre for Disease Prevention and Control,^8^ plots of the daily global numbers of COVID-19-infected persons during the second trimester of 2020 reveal an undulating and ascending pattern that is difficult to explain. However, they also show a progressive and steady decrease in mortality for that same period.^7^ This last observation merits a logical explanation, but there has not been enough research to date to provide one. The cumulative number and type of mutations detected during the first 6 months of 2020 do not explain the reduced severity of SARS-CoV-2.^50^ Table 4 also demonstrates the predominance of RNA viruses as the cause of most pandemics, with the only exception being the variola virus, which is composed of DNA. Relatively few pandemics were caused by Gram-negative bacteria.

### Pandemics Originating Mainly in Tropical Areas

Yellow fever epidemics occur mainly in Africa and South America. The disease is transmitted by several species of mosquitoes, mainly by *Aedes aegypti*. The disease life cycle has two possible scenarios: (1) the sylvatic cycle, where the hosts are various types of animals, primarily monkeys; and (2) the urban cycle, where the reservoir is human beings. Outbreaks occur mainly during the rainy season. Water accumulation, on land or in various objects, facilitates development of vector mosquitos. Other viruses transmitted by *Aedes aegypti* include dengue virus, Zika virus, and chikungunya virus.^34^

For close to half a century, several Ebola virus outbreaks have occurred in Western and Central Africa. The long-term reservoir of this virus is a species of bats. Due to massive deforestation, there have been episodes of viral spillover to humans and animals, including gorillas and chimpanzees. Subsequently, human-to-human transmission occurred as the result of close contact with patients or bodies at burial ceremonies, and some convalescing patients continued to transmit the virus for some time after recovery. Due to the high contagiousness of Ebola, many health care workers were also infected. Currently, there is an effective vaccine to prevent Ebola (Table 5) as well as a drug composed of three types of antibodies (Table 6).^51^

**Table 5:** Vaccines for Pandemic Diseases.*

Disease	*R_0_*	Vaccine(s)	Current Number of Cases/Year
Smallpox	3.5–6	Yes†	None
Measles	12–18	Yes†	5M
Plague	‡	Yes†	0.5K
Cholera	‡	Yes†	1.4–4M
Yellow fever	‡	Yes§	200K
Influenza H1N1	1.4–2.8	Yes†	>5M; various types of influenza
Influenza H3N2	1.5	Yes†	>5M; various types of influenza
Influenza H2N2	1.5	Yes†	>5M; various types of influenza
AIDS	‡	None	1.7M
SARS	0.19–1.08	None	None
Ebola	1.5–1.9	Yes†	0–several thousands
MERS	0.3–0.8	None	0–0.3K
COVID-19	2.5	None	>10M**

* Only approved vaccines are included.

† Various types available.

‡ Difficult to assess.

§ Live attenuated vaccine, single shot gives life-time immunity.

** During a 6-month period.

AIDS, acquired immune deficiency syndrome; COVID-19, coronavirus disease 2019; H1N1, hemagglutinin-1 neuraminidase-1; H2N2, hemagglutinin-2 neuraminidase-2; H3N2, hemagglutinin-3 neuraminidase-2; K, thousands; M, millions; MERS, Middle East respiratory syndrome; *R_0_*, basic reproduction number; SARS, severe acute respiratory syndrome.

**Table 6:** Treatment Used for Pandemic Diseases.*

Disease	Treatment
Smallpox	Tecovirimat†
Measles	Vitamin A
Plague	Doxycycline‡
Cholera	Massive rehydration; doxycycline (in adults)‡; azithromycin (in children)‡
Yellow fever	None
Influenza H1N1	Oseltamivir§
Influenza H3N2	Oseltamivir§
Influenza H2N2	Oseltamivir§
AIDS	>20 antiviral drugs grouped in 8 classes and >40 combinations of two or more drugs
SARS	None
Ebola	REGN-EB3**
MERS	None
COVID-19	Remdesivir††

* Only approved drugs are included.

† Never used in humans. Two million doses stockpiled in the US, in case of bioterror attack.

‡ Other effective antibiotics available.

§ Other effective antivirals available.

** Includes three types of antibodies.

†† Several other antivirals under investigation.

AIDS, acquired immune deficiency syndrome; COVID-19, coronavirus disease 2019; H1N1, hemagglutinin-1 neuraminidase-1; H2N2, hemagglutinin-2 neuraminidase-2; H3N2, hemagglutinin-3 neuraminidase-2; MERS, Middle East respiratory syndrome; SARS, severe acute respiratory syndrome.

### Influenza Pandemics

During the twentieth century, there were four major pandemics: H1N1 caused the 1918 influenza pandemic; the pandemics of 1957, 1958, and 2009 were all descendants of the 1918 virus. The last of these pandemic influenza viruses incorporated genes by reassortment.

In general, annual seasonal influenza in post-pandemic years is caused by variants of the corresponding virus from the prior pandemics. Influenza viruses involved in seasonal flu accumulate antigenic changes in a progressive fashion resulting in annual seasonal epidemics. An accepted parameter for the impact of annual influenza activity is the excess number of deaths.^52^,^53^ Interestingly, in 1977, a re-emergence of human H1N1 viruses identical to those circulating before 1957 was attributed to an accidental “escape” of an old frozen laboratory specimen.

## THE IMPACT OF PANDEMICS ON HUMAN HISTORY

Epidemics and pandemics have had a very strong impact on human history. Diseases like smallpox, measles, and plague decimated entire populations in several regions of Europe, the Middle East, and Asia. At the end of the fifteenth century, the European conquistadores of the Americas brought diseases with them that were unknown to the native population. Due to the local lack of immunity to these newly imported viruses, smallpox and measles spread rapidly, causing fear and frustration among the natives due to the lack of natural resistance and a very high mortality rate; local communities were decimated and sometimes entire settlements were wiped out. This tragedy facilitated the conquest of the land and the massive conversion of Indian tribes to Christianity across the Americas.^34^

Beginning in the last decade of the twentieth century, the AIDS pandemic intensified its spread on all continents, inflicting the greatest damage in Africa at the social, economic, and political levels. Some of the manifestations of this megacatastrophe were the significant shortening of life expectancy, massive destruction of family units, and orphanhood. In addition, the profound immune suppression caused by HIV led to a rampant increase in the incidence and prevalence of tuberculosis and other infectious diseases. Combined, these factors had a disastrous effect on the political structure of most African countries.^34^,^35^

Across history, pandemics have differentially infiltrated battling armies and in this way tipped the outcome of battles and war.^34^

## PALEOMICROBIOLOGY

During the last decades and as a valuable complement to the written documentation on ancient bacterial pandemics, paleomicrobiological studies relating primarily to the plague (*Yersinia pestis*) have contributed greatly to the reshaping of our understanding of its epidemiology. There was much confusion and controversy regarding the epidemiology of plague in the multiple pandemics that occurred several centuries ago, until the studies of French investigators, Drancourt and Raoult and colleagues, which revolutionized several contentious concepts established by other investigators.^54^ Their work centered on the skeletal remains of common graves from various epochs.^54^–^57^ By examining the bacterial DNA in the pulp of relatively preserved teeth, they found genomic evidence for not only *Yersinia pestis* but also for *Bartonella quintana*, a bacterium transmitted by lice.^54^,^57^ Since *Yersinia pestis* is also found in body lice, the investigators developed a laboratory rabbit model and exposed the animals to human lice infected with specific bacteria. Additional epidemics investigated by the same scientists were found to be caused by *Bartonella quintana* alone or combined with *Rickettsia prowazekii*.^54^ These and other studies supported the theory that infected lice were key spreaders of *Yersinia pestis*, sometimes found in polymicrobial infections.

With regard to smallpox, several well preserved mummies have been found in Egypt and other countries. Some mummies, such as that of the Egyptian pharaoh, Ramses V, provided dermal evidence that they had terminally suffered from smallpox. However, genomic evidence for the actual virus has been found only rarely in mummies. This can be explained by the poor long-term preservation of viruses. Other diseases documented by paleomicrobiological methodologies include tuberculosis, leprosy, and multiple parasitic infections.^54^

## TRACING THE ORIGIN OF MICROBES AND THEIR ROUTE OF TRANSMISSION

**Figure 3:**
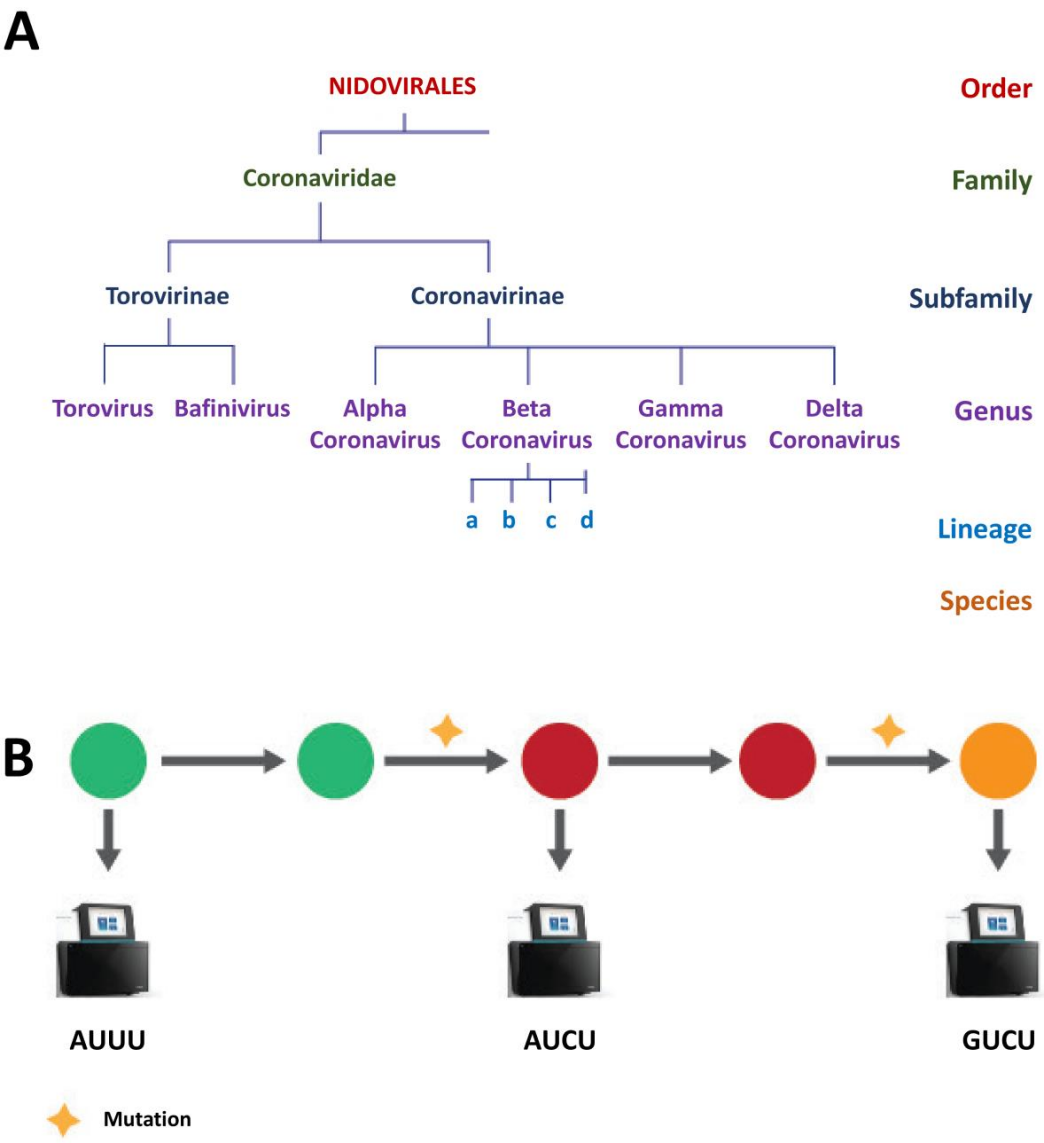
Genomics as a Useful Tool in Pandemics **A:** Sequencing of representative isolates to create a phylogenetic tree of a coronavirus. **B:** Tracing a pandemic pathway. Specific viral isolates are sequenced to detect mutation and thereby reconstruct the chain of transmission. In panel **B**, each circle represents a coronavirus isolated from a patient. Different colors indicate a virus mutation. A genome sequencer device (machines in the figure) is used to determine the location and type of mutation: AUUU, a segment of the viral genome Adenine Uracil Uracil Uracil; AUCU, same segment of the genome after mutation Cytosine replacing middle Uracil; or GUCU, same segment of the genome following another mutation: Guanine replaced Adenine.

The past 30 years have seen rapid development of genetic sequencing technologies for RNA and DNA.^58^ From the beginning, RNA/DNA research has been characterized by both exaggerated promises and inflated expectations regarding the importance of human genetic traits that predispose for both infectious and non-infectious diseases.^59^ Unfortunately, to date, no human genetic markers predisposing to SARS-CoV-2 infection, nor the severity of COVID-19, have been found—although recent isolated exceptions to this statement can be found. For example, there may be a predisposition to COVID-19 among humans with blood group A as opposed to other blood groups.^60^ In sharp contrast to human genomics, mapping viral RNA mutations of SARS-CoV-2 has enabled a fairly accurate reconstruction of its transmission (Figure 3B) and helped to determine its phylogenetic origins (Figure 1 and Figure 3A).^1^,^61^ Viral sequencing of samples obtained from COVID-19 patients, and being able to mark its different mutations, has provided a much clearer and more accurate picture of viral transmission as opposed to results obtained by contact tracing. For example, a study of disease importation to continents, countries, or regions has surprisingly uncovered several shuffled patterns in the genomic data. When considering importation of the first COVID-19 cases to the Western United States, the initial investigation based on contact tracing indicated that those cases were epidemiologically unrelated. However, after sequencing multiple virus isolates, the researchers concluded that they were not only closely related, but probably also began with one patient of origin.^62^

A series of questions must be answered when attempting to determine the primary source of a pandemic virus like SARS-CoV-2. As it is a coronavirus, the most probable source is a virus that originated in bats.^63^ To confirm this theory, genomic profiles of coronaviruses previously isolated from bats must be compared with coronavirus profiles isolated from patients. If these sequences are identical, then it can be assumed that the primary source is a bat.^64^ However, if the virus has been preserved in the refrigerators of a virology laboratory, there exists a very real possibility of viral “escape” from the laboratory to the community. In this scenario, especially if the laboratory is located in the proximity of the epidemic epicenter, a laboratory origin of the pandemic virus would be a strong alternative. Additional tests such as antibody testing of the personnel working in the suspected laboratory should provide further assistance in elucidating the true origin and pathway of the virus.

Other possible trajectories should also be investigated. For example, if early infections occurred in persons who had close contact to bats or other animals, such as animals raised on farms or held and sacrificed at wet markets, this would clearly point to a specific zoonotic link. Another possibility is that the new virus was engineered for investigational purposes or with malicious criminal intentions. By looking at the structural details of the virus, molecular biologists have ruled out and published the arguments against this last-mentioned possibility.^65^

## MEASURING PANDEMICS

The COVID-19 pandemic exemplifies the difficulties encountered when attempting to quantify the important numerical parameters of the disease. First of all, when trying to count the number of infected cases, this parameter is significantly underestimated, primarily because the proportion of people who are asymptomatic or suffering from mild disease are not yet known.^66^ Secondly, the initial shortage of laboratory kits has limited the number of people who can be ideally tested.^67^ The same problem arises when attempting to calculate the basic reproduction number, i.e. the number of persons who were infected by a certain patient. In this case, it is not easy to draw a line to those who should be considered a contact.^68^ A similar bias can interfere with calculation of the case fatality ratio, i.e. the proportion of infected persons who have died of COVID-19. In addition to underdetection of mainly asymptomatic patients, some deaths due to COVID-19 complications passed under the radar since the patients had not been tested for the virus. Last but not least, the sensitivity and specificity of viral tests are not 100%, resulting in occasional false negative or false positive results.^69^ A complementary method to assess pandemic-related mortality is to measure excess deaths compared to previous years during the same season.^70^ In order to retrospectively estimate the number of infected persons, serologic tests are performed that measure the presence of specific IgM and IgG antibodies to SARS-CoV-2. Currently, there are many ongoing seroprevalence studies of anti-SARS-CoV-2 antibodies; however, the picture remains incomplete regarding the significance of these findings as markers of previous infections.^71^

## CONTAINMENT OF EPIDEMICS AND PANDEMICS

In 1854, a cholera outbreak erupted in the densely populated neighborhood of Soho, London. John Snow, a physician who lived in the vicinity, mapped the houses of the many affected persons with cholera. He discovered a water pump in the center of the mapped area that had been used by all infected persons for obtaining drinking water. On his advice, municipal authorities removed the pump handle, resulting in an immediate end to the epidemic, with no further cases. It was subsequently learned that human excrement pits had drained into and contaminated the water supply.^72^ This brilliant epidemiological investigation led to John Snow being considered the “Father of epidemiology.”^73^

In the 1990s a major epidemic of cholera occurred in Peru. *Vibrio cholera* contaminated the ballast water in ships that had arrived from India. Massive contamination of large quantities of live fish occurred when the ballast water was discharged into the sea near the shore. Fish were subsequently caught from this area, and thousands of Peruvians developed cholera after consuming raw fish (ceviche), a popular traditional food. The epidemic was terminated abruptly when the health authorities recommended to discard the head and branchia (gills) when preparing the fish for consumption.^74^

Following the spread of the COVID-19 pandemic, the health authorities of most countries imposed a lockdown with various intervals of delay from the first detected case(s) so as to contain the local spread of the virus. Subsequently, they also imposed the use of respiratory masks to reduce airborne transmission by infected persons and to prevent contagion among the uninfected population. Data from several countries have shown that the earlier and more stringent the lockdown was applied, the better the efforts in containing the pandemic.^75^

### Vaccines for Prevention of Pandemic Diseases

Vaccines are human-made molecular tricks aimed at cheating the immune system of the host, to make it “believe” that it has encountered a microorganism-causing disease (Professor Myron Levine, personal communication). Table 5 lists the current availability of approved vaccines for pandemic diseases. Paradoxically, some of the newer diseases such as AIDS and those caused by coronaviruses still lack an approved effective vaccine. On the other hand, smallpox, a very old but already eradicated disease, was (to the best of our knowledge) the first disease for which several types of “natural” empiric vaccines were used, initially by the Chinese in ancient times,^76^ and subsequently by Edward Jenner in England in the eighteenth century.^76^,^77^ Interestingly, vaccination with smallpox vaccines was implemented several centuries before the discovery of viruses in general, and variola and vaccinia viruses in particular.

As recently summarized by the WHO,^78^ there are currently at least 125 different COVID-19 vaccination research projects underway in the quest to prevent COVID-19. Essentially, six groups of vaccines are being explored, three of which first require the isolation of viral particles. These virions are either: (1) weakened (attenuated); (2) killed by hot or chemical substances (inactivated); or (3) fragmented viruses followed by isolation of small pieces of the virus (subunits). A second class of vaccine starts with genetically engineered pieces of either RNA or DNA, which subsequently are embedded in either: (4) plasmids (DNA); (5) lipids (RNA); or (6) an adenovirus vector.^79^ In all cases, particles are diluted in a solution for initial animal testing (preclinical trials) and subsequent testing in humans (clinical trials).

Clinical trials are performed in three phases, each one having an increasing number of volunteers. Phase I (10–100 volunteers) examines safety and immunogenicity. Phase II (>100 volunteers) examines safety, immunogenicity, and dose adjustments. Phase III (more than 10,000 volunteers) primarily examines infection prevention following exposure. If all three phases are successful, then the vaccine undergoes regulatory approval and subsequent mass production under strict quality control.

The main problem with the need to develop a vaccine against a new pandemic microbe is that the process is lengthy and mined with multiple obstacles.^80^,^81^ In the past, the elapsed time from vaccine development to approval and production could take up to 10 years. However, in order to efficiently and rapidly cope with an emerging pandemic, ideally, the scientific world must be prepared with predefined “templates” to facilitate accelerated vaccine development. If a proposed vaccine causes serious side effects or alternatively is not sufficiently immunogenic, the development process should be restarted from the beginning. In some cases, when a clinical evaluation of a vaccine reaches Phase III, the number of newly infected cases drops rapidly, making it difficult to test the actual efficacy of the vaccine in preventing infection. In the quest for a COVID-19 vaccine, there have been a few initiatives to recruit volunteers who would be challenged with the virus after being immunized. However, for this scheme to work, it is essential to have a very efficacious drug available for treating the infection, should the vaccine fail.

### Drugs to Treat Pandemic Diseases

In general, with regard to the efficacy of antiviral treatments, viral infections can be divided into three groups: (1) infections lacking an effective antiviral therapy (e.g. SARS, yellow fever, measles); (2) infections in which antivirals do not cure the infection, but which do produce varying degrees of clinical improvement (e.g. influenza, AIDS, COVID-19); and (3) infections that can be cured by antiviral therapy (e.g. Ebola, hepatitis C). Table 6 provides an overview of the treatments used for different pandemic diseases.

Despite an increase in antibiotic resistance, there remain multiple choices for treatment of bacterial pandemics. For example, the main pillar of medical treatment for cholera is the emergency replacement of large amounts of fluid lost as a result of diarrhea and vomiting, with antibiotics playing a secondary role in its treatment. The fatality ratio of untreated cholera is around 50%. However, prompt administration of appropriate amounts of fluids either orally or intravenously decreases the case fatality ratio to 1%.^48^

The overwhelming death toll from COVID-19 has sparked a myriad of projects to identify drugs that can be repurposed on a fast track to for special treatment of patients with severe disease.^6^ In the meantime, the only drug that has shown some beneficial results in a double-blind randomized clinical trial compared to placebo is the antiviral remdesivir, which has been approved by the US Food and Drug Administration and other regulatory institutions.^82^

The administration of convalescent plasma from recovering patients with COVID-19 is now being examined at different sites, including new clinical trials, but conclusions regarding this therapy are still pending.^83^

## ECONOMIC IMPACT OF COVID-19

The economic impact of past pandemics is hard to examine due to the lack of robust data.^84^ However, a retrospective look at the first 6 months of the COVID-19 epidemic reveals a catastrophic impact on the economies of most countries having to cope with significant numbers of cases. The harshest economic impact generally occurred in varying degrees in wealthier countries. One of the most important parameters for quantifying economic damage is the gross domestic product forecast, although different countries use other economic metrics. According to the majority of prognoses, the damage was expected to be greatest during the second quarter of 2020. The primary reason for the severe economic impact of COVID-19 has been the leading and widely justified slogan, “health before wealth.” Another parameter that has significantly contributed to the economic crisis worldwide is the swift increase in job losses. Quantification of financial activities in selected populations may add important data to more accurate evaluation of the world economy as a result of the COVID-19 pandemic. For example, listing the purpose of out-of-home visits (residential, parks, workplace, grocery stores, pharmacy) may contribute to a composite financial evaluation of representative family units.^84^,^85^

## CONCLUSION

Despite the rapid and advanced progress in many medical disciplines since the end of the nineteenth century, the COVID-19 pandemic has sadly demonstrated vast limitations worldwide in successfully coping with it. There is no doubt that the unexpected and yet fully unknown behavior of SARS-CoV-2 has strongly contributed to its pandemic status. For example, the high proportion of infected but totally asymptomatic persons has made containment challenging, to say the least. In some cases, the proportion of infected persons feeling absolutely well can approach almost 90%.^86^ For example, a COVID-19 outbreak occurred on a cruise ship departing Ushuaia in the province of Tierra del Fuego, Argentina, and navigating to the Antarctic peninsula. Sampling the entire population on board revealed that a vast majority had contracted the infection, but 80% of them were asymptomatic patients.^86^ Another tricky characteristic of the virus is that infected patients expelled virus particles through their respiratory tract, primarily during the early phases of the incubation period before they became symptomatic.

Selected examples from prior pandemics should illuminate our vision for the future. Smallpox went from being a totally empiric vaccine to global eradication of the disease. However, AIDS, which is caused by a zoonotic retrovirus that translates its RNA to DNA and enters the human genome, presents an almost impossible challenge in approaching a total cure for the disease, although combinations of antivirals are able to halt or reverse the progression of the disease.

This retrospective analysis and comparison of COVID-19 with prior pandemic diseases can contribute to the improvement of a rationale and scientific approach to future epidemics or pandemics. The most important take-away point should be an understanding of the high degree of preparedness that is needed, including various protocols for social distancing that are adapted to the different transmission modalities of the involved microbes. In addition, multiple innovative protocols aimed at a robust accelerated vaccine development process are needed. Disease-causing viruses, or colonizing species in the animal kingdom, should be evaluated for potential spillage to human beings. For those specific viruses, it is imperative to delineate seminal protocols that can be launched in emergency situations.

Recently, the New York times launched two very recent dynamic applications that allow tracking the daily status of 20 therapies and 155 vaccines for COVID-19.^87^,^88^ Readers can visit these sites to access updates regarding ongoing developments related to COVID-19 vaccines and treatments.

Finally, the conclusion of this paper, at this point in time, is to stress the importance of ongoing refinement of interactions between government leaders, scientists, and economists, at both the national and international levels, so as to better grapple with the current (and any future) pandemic, as it unfolds.

## Abbreviations

AIDS: acquired immune deficiency syndrome
COVID-19: coronavirus disease 2019
DNA: deoxyribonucleic acid
Flu: influenza
gRNA: guide ribonucleic acid
H1N1: hemagglutinin-1 neuraminidase-1
H2N2: hemagglutinin-2 neuraminidase-2
H3N2: hemagglutinin-3 neuraminidase-2
MERS: Middle East respiratory syndrome
MERS-CoV: Middle East respiratory syndrome coronavirus
RNA: ribonucleic acid
SARS: severe acute respiratory syndrome
SARS-CoV-2: severe acute respiratory syndrome coronavirus-2; spp, species
US: United States
WHO: World Health Organization.
